# Supporting factors and structural barriers in the continuity of breastfeeding in the hospital workplace

**DOI:** 10.1186/s13006-022-00533-1

**Published:** 2022-12-19

**Authors:** Paveewan Jiravisitkul, Saraiorn Thonginnetra, Naruporn Kasemlawan, Thitiphong Suntharayuth

**Affiliations:** 1 Department of Pediatrics, Chulabhorn Hospital, Chulabhorn Royal Academy, Bangkok, Thailand; 2 Data Management Unit, Centre of Learning and Research in Celebration of HRH Princess Chulabhorn’s 60th Birthday Anniversary, Chulabhorn Royal Academy, Bangkok, Thailand

**Keywords:** Breastfeeding, Supporting factors, Barrier, Employee, Hospital workplace, Working mother, Returning to work

## Abstract

**Background:**

The World Health Organization recommends that babies should receive exclusive breastfeeding (EBF) for six months, and mothers should be encouraged to breastfeed until their infant is aged two years or older. The breastfeeding rate in Thailand is currently much lower than the target. One critical factor is lactating mothers returning to work, especially in a hospital workplace with high job stress. In this study, we aimed to identify supporting factors and obstacles to sustaining breastfeeding in hospital-type workplaces.

**Methods:**

We conducted a mixed methods study between February 2021 and August 2021 at Chulabhorn Hospital, Thailand. Quantitative data were collected using questionnaires, and qualitative data were gathered in a focus group discussion among purposefully chosen participants, including mothers with both successful and unsuccessful continuation of breastfeeding after returning to work. We conducted multivariate analysis and thematic analysis in quantitative and qualitative data analysis, respectively.

**Results:**

Questionnaires were completed by 65 permanent employees of the hospital, and seven of these participated in focus group discussion. The rate of exclusive breastfeeding from birth to six months was sixty six percent, and was measured by the responses from questionnaires, which defined as a practice whereby the infants receive only breastmilk without mixing it with other food. Seventy-seven percent of participants were continuing to breastfeed when they returned to work, with 24% (12/50) having to stop after three months. Factors associated with exclusive breastfeeding was caesarean delivery (OR 7.9; 95% CI 2.00, 31.08). Additionally, childcare at the workplace and the attitude of colleagues were found to be supporting factors for sustaining breastfeeding. Barriers included inadequate private facilities (location and equipment), lack of a breastfeeding break, workload, and inconvenient uniform.

**Conclusions:**

Effort is needed to sustain breastfeeding after maternal return to work. Our findings are crucial in determining how best to support nursing mothers in breastfeeding after returning to work, particularly during the ongoing COVID-19 pandemic. A breastfeeding-friendly policy with clear operating guidelines in the workplace is critical to sustaining breastfeeding. Learning from others who have had a positive experience will ensure that all breastfeeding women are better supported in the workplace in future.

## Background

Currently, there is much scientific evidence confirming the benefits of breastfeeding for both infants and mothers [1]. The World Health Organization (WHO) and the United Nations Children’s Fund (UNICEF) have set forth recommendations for breastfeeding: babies should be breastfed within one hour after birth, should receive exclusive breastfeeding (EBF) for six months, and mothers should breastfeed with complementary feeding until the infant is aged two years or older [2, 3]. The target rate for EBF is 50% by 2025 [4], but current data show that the rate in Thailand is currently much lower than the target [5]. Several factors contribute to successfully reaching the EBF goals, including maternal and infant health factors, maternal attitude toward breastfeeding, hospital breastfeeding promotion policies, family support, and the societal context and beliefs including regarding breastfeeding mothers returning to work [6].

Maternal return to work before the infant’s age six months is very challenging for continuous breastfeeding. Therefore, the collaboration of private and public sectors is very important. To achieve that goal, Thailand legally extended maternity leave from 90 to 98 days in May 2019 [7], but it has not yet been specified to employers to provide a breastfeeding room in the establishment and include the breastfeeding break during the work hours [8]. Nevertheless, the maternal leave allowance in Thailand is less than six months after delivery; therefore, workplace support is an important part of the success rate of EBF for six months. Several studies have found that mothers who must return to work have a nearly two-fold lower rate of EBF than those who do not [9, 10]. The reasons for shortened duration of breastfeeding may be the difficulty of managing both breastfeeding and work [11]. Factors that hinder breastfeeding at work include a conservative workplace, being domiciled in another province, and the short maternity leave [12]. Another major obstacle is a workplace environment that is not conducive to expressing breast milk, such as not having a place to express and store breast milk [13]. Moreover, expressing breast milk is time-consuming and might interfere with work, so an understanding attitude among supervisors and colleagues is essential [14].

In hospital workplaces of Thailand, healthcare workers have high job stress [15, 16], so continuing breastfeeding after returning to work requires support from all sectors. Regarding the EBF rate among hospital employees, workers in public hospitals had higher breastfeeding rates than those in private hospitals [17]. The private spaces for breastfeeding or expressing milk, support from supervisors and co-workers, flexible scheduling and providing breast pumping equipment are important factors that affect continuous breastfeeding among mothers returning to work [18, 19]. In Thailand, the situation of breastfeeding women employed by a hospital has not been investigated, as well as workplace supports available for breastfeeding women to meet their needs.

Therefore, in this study, we aimed to identify the supporting factors and structural barriers at work for continuous breastfeeding after maternity leave, in a hospital-type workplace. Understanding these factors will lead to the design of hospital workplaces that are conducive to breastfeeding for working mothers, including the creation of policies, facilities, environments, and corporate cultures that facilitate sustained breastfeeding.

## Methods

### Study design and population

As we aimed to identify the supporting factors and structural barriers at work for continuous breastfeeding in Thai healthcare workers which had never been explored, thus we used both quantitative and qualitative approaches, namely mixed methods design, to understand the “what and why” of the situation [20]. The research study conducted between February 2021 and August 2021 at Chulabhorn Hospital, Chulabhorn Royal Academy in Bangkok, Thailand. The explanatory sequential design was divided into two phases. We conducted a cross-sectional descriptive study and collected data using a structured online questionnaire to explore the employee’s maternal and infant characteristics, maternal work condition, attitudes toward breastfeeding and breastfeeding habits, breastfeeding rates before and after returning to work, and availability of supporting modalities in the workplace. Then, we selectively sampled participants based on their responses and followed up a number of factors we found from quantitative data qualitatively via an online guided focus group discussion (FGD).

### Sample size determination and sampling procedures

The study population was female employees who were permanent employees of Chulabhorn Hospital and whose maternity leave was scheduled while being an employee of the hospital. The inclusion criteria were maternity leave scheduled to begin between December 1, 2018 and November 30, 2020; and working at Chulabhorn Hospital for at least one month on the day of participation in the research study. Mothers whose child had died before the age of six months were excluded. Collecting data through questionnaires could lead to a recall bias, and we decided to gather data in the last two years. Thus, out of the 1,113 total female employees, 84 of them took maternity leave during those 2-year period.

In qualitative sampling, we used a purposive sampling method to select participants who were successfully breastfeeding before returning to work from their interesting questionnaire responses, for example, participants who rated the breastfeeding support in workplace with the lowest score (1) or highest score (5), participants with short maternity leave but were able to successfully continue breastfeeding, or participants who showed a strong willingness to breastfeed in questionnaire responses but were unable to continue breastfeeding. We divided the selected participants into a group that was able to continue breastfeeding successfully after returning to work, and a group that was unable to continue breastfeeding, defined as breastfeeding stopped within six months of returning to work. Seven study participants from the total sample of 65 were included in the FGD.

### Operational definition

*Exclusive breastfeeding* defined as a practice whereby the infants receive only breastmilk without mixing it with other milk or food during the first six months of life. The EBF rate was measured by the responses of participants from questionnaires, which reported by individual participant regarding their self-assessment on breastfeeding practice.

The questions list is

‘*Did you exclusively breastfeed for 6 months?’*

*‘If not, did you give mostly infant formula or breastmilk for the first 6 months?’*

*‘How long did you exclusively breastfeed?’*

*‘Please specify the age of your child that infant formula/whole milk was initially given’*

*‘At what age of your child that you stopped breastfeeding?’*

### Data collection

In the quantitative analysis, we sent an online questionnaire via email to 84 female employees of Chulabhorn Hospital who met the inclusion criteria. Detailed information and an electronic informed consent form were included in the email. All information was kept confidential and was used in this research only. One month later, we collected quantitative data from participants’ responses to the questionnaire.

In the qualitative analysis, we recruited seven participants for the FGD, divided into subgroups of successful (*n* = 4) and unsuccessful (*n* = 3) continuous breastfeeding after a return to work. The FGD was conducted using Google Meet and lasted for approximately 90 min. The discussion was facilitated by a team of three researchers by using the FGD guide. The FGD guide was used to collect information on the factors that affected the continuation of breastfeeding in the hospital workplace. The FGD guide was developed in Thai and pre-tested for clarity among three women employees at Chulabhorn Hospital before conducted the FGD. One researcher acted as the discussion leader assisted by two researchers who acted as a co-facilitator and an observer, respectively. The researcher first introduced ourselves and explained the objectives of the FGD. The interview divided into four main sessions, which started with the breastfeeding intention during prenatal period, followed by the breastfeeding experiences during maternity leave and the third part focused on breastfeeding experience after returning to work. The last session of the meeting, the interviewer was open to attendees to summarize the key success factors, common obstacles and solutions. After the FGD, the researchers met to summarize the key points of the discussion.

#### Quantitative data analysis

Descriptive data are reported as frequency and percentage, mean ± standard deviation (SD), or median and interquartile range (IQR) to describe the sample. We used chi-squared test or Fisher’s exact test for categorical variables, and the Student *t*-test or Mann–Whitney U test (if data were not normally distributed) for continuous variables, to investigate factors affecting successful breastfeeding between groups that were able to continue breastfeeding and those that stopped breastfeeding within six months because of returning to work. Further, binary logistic regression was used to determine the likely predictors of exclusive breastfeeding, starting with the univariate logistic regression analysis follow by the multivariate logistic regression analysis. The multivariate analysis was used to assess the independent of explanatory variable on exclusive breastfeeding, all co-variates with a *p* value < 0.05 in the univariate logistic regression analysis were taken in the multivariate analysis. The results were presented using adjusted odds ratio, and confidence intervals. Stata version 16 was used to analyze the data.

#### Qualitative data analysis

The FGD was conducted using an online platform, a recorded online meeting was used for further analysis. Thematic coding analysis was used to analyze the qualitative data using first-level open coding and second-level coding to group the initial codes into a small number of themes [21]. Participants’ responses were transcribed verbatim manually. We verified each transcript with memos taken both during and right after the interview. Initially, parts of the interview transcripts were coded independently by ST and PJ, then we openly talked about each coded section’s interpretation of its intended meaning. However, different researchers occasionally used different phrasing to explain how they interpreted the transcription. We, ST, PJ and NP then revisited the transcription and memo before making a team agreement. After coming up with the initial coding, we had developed a working analytical framework and a matrix summarizing the data for each theme.

## Results

### Descriptive results

A total of 65 of the 84 selected participants provided complete responses to the questionnaire. The demographic data of mothers and infants are shown in Table 1.

**Table 1:** Demographic data of mothers and their infants (*N* = 65)

**Maternal characteristics**	**n (%)**
Maternal age (y), mean (standard deviation)	33.98 (4.11)
Parity	
1	44 (68)
More than 1	21 (32)
Educational level	
High school	7 (11)
Bachelor’s degree	46 (71)
Master’s or higher	12 (18)
Income (Baht per month)	
Less than 20,000	14 (22)
20,001–50,000	34 (52)
More than 50,000	17 (26)
Marital status	
Married	62 (96)
Other	3 (4)
Underlying diseases or complications during pregnancy	12 (19)
**Infant characteristics**	**n (%)**
Infant age	
6–12 months	14 (22)
1–2 years	31 (48)
More than 2 years	20 (30)
Birthweight (g), median (interquartile range)	2950 (2535–3365)
Gestational age (weeks)	
35–37	13 (20)
38–40	52 (80)
Infant illness during the first 28 days	9 (14)
Delivery type	
Vaginal delivery	18 (28)
Cesarean delivery	47 (72)
Delivery hospital	
Public hospital	51 (78)
Private hospital	14 (22)

Participants included the following professionals: doctor or dentist (25%), nurse (25%), nursing assistant (18%), and others including general staff, pharmacist, occupational therapist, medical technologist, academician, teacher, and babysitter (32%). The average duration of maternity leave was 95 days. Ninety percent of participants had worked in Chulabhorn Hospital for more than two years (Table 2).

**Table 2:** Work information of participants (*N* = 65)

Variables	n (%)
Duration of work	
Less than 2 years	4 (6)
2–5 years	33 (51)
More than 5 years	28 (43)
Average maternity leave (days), mean (standard deviation)	95 (90)
Maternity leave	
Less than 90 days	29 (45)
91–120 days	17 (26)
More than 120 days	19 (29)
Occupation	
Doctor/dentist	16 (25)
Nurse	16 (25)
Nursing assistant	12 (18)
Other	21 (32)
Number of working hours per week	
Less than 41	31 (48)
41–45	15 (23)
More than 45	19 (29)
Working time characteristics^a^	
Working regular hours	35 (54)
Work shifts	30 (46)

^a^’Working regular hours’; work during weekday from 8.00 am to 4.00 pm

‘Work shifts’; Day shift (8.00 am-4.00 pm) Evening shift (4.00 pm-12.00 pm) and Night Shift (12.00 pm-8.00am), Number of working hours range from 40–70 h per week

Attitudes toward breastfeeding and breastfeeding habits are shown in Table 3. Approximately 90% of participants intended to EBF for six months and made prenatal breastfeeding preparations. All mothers in this study had prepared breastfeeding equipment, such as breast pumping equipment and a nursing cover which is a piece of fabric draped over the breast to provide privacy when breastfeeding in public. The average stress score from self-rated stress score was 3.28, with the three leading causes of stress being parenting (37%), breastfeeding (35%), and family pressure (15%). Eighty-five percent (55/65) of participants chose to give birth in a hospital that supports breastfeeding, but only 55% (36/65) received information from the hospital related to breastfeeding. Only 20% (13/65) were able to breastfeed within one hour after birth (Table 4).

**Table 3:** Attitudes toward breastfeeding and breastfeeding habits (*N* = 65)

Variables	n (%)
Intention to exclusively breastfeed	58 (89)
Prenatal preparations for breastfeeding	60 (92)
Previous lactation experience	21 (32)
Parenting assistant during maternity leave	46 (71)
Supported by family members	62 (95)
Method of breastfeeding during maternity leave (*n* = 63)	
Direct breastfeeding	53 (84)
Breast pumping only	10 (16)
Average stress scores (range 1–5), mean (standard deviation)	3.28 (1.19)

**Table 4:** Rates of breastfeeding before and after returning to work

Variables	n (%)
Infant breastfed within 1 h after birth (*n* = 65)	13 (20)
Exclusive breastfeeding for 6 months (*n* = 65)	43 (66)
Breastfeeding status before returning to work (*n* = 65)	
Stopped	15 (23)
Still breastfeeding	50 (77)
Stopped breastfeeding after returning to work (*n* = 50)	12 (24)
Within 1 month	5/12 (42)
1–2	5/12 (42)
2–3	2/12 (17)

The rate of infant breastfed within one hour after birth was 20% (13/65), which was 21% (10/47) in the caesarean group and 17% (3/18) in the vaginal delivery group (*p* = 1). The rate of EBF in this study was sixty-six percent. Seventy-seven percent of participants were continuing to breastfeed before they returned to work, with 24% (12/50) having to stop breastfeeding within three months after returning to work (Table 4). The main reasons for discontinuing breastfeeding at work included no break for pumping milk (27/50, 54%), no place to express milk (25/50, 50%), excessive working hours (13/50, 26%) and distance between home and the workplace (9/50, 18%); twenty-four percent (12/50) of participants said there were no barriers at work. Among participants who were able to continue breastfeeding at work for more than three months (*n* = 38), obstacles to continued breastfeeding at the workplace were a lack of policies supporting breastfeeding (9/38, 24%), the number of maternity leave days (6/38, 16%), and the attitudes of colleagues and supervisors (4/38, 11%). Most participants (72%) commented that their workplace did not have a policy to support breastfeeding, including experts in counseling and facilities for pumping and milk storage (Table 5).

**Table 5:** Responses from participants regarding the workplace and breastfeeding (*N* = 65)

Variables	n (%)
Policies that support breastfeeding in the workplace	18 (28)
Breastfeeding specialists or consultation teams in the workplace	16 (25)
Appropriate duration of maternity leave	50 (77)
The workplace has a breastfeeding room	9 (14)
Allowed to express breast milk during work time	40 (62)
The workplace has a refrigerator used for storing expressed milk	24 (37)

Nearly 40% of mothers (23/59) wanted to send their infant to childcare, but only half (12/23) were able to do so. Seventy-five percent (9/12) of participants who sent their infant to childcare found that childcare played a supporting role in sustained breastfeeding. Among women who did not work far from the center and had sufficient break time to visit the center during the day, they reported that they could breastfeed during the day at the daycare center.

Bivariate analysis showed that the factors associated with EBF for six months are delivery type, duration of maternity leave, occupation, and having breastfeeding specialists or consultation teams in the workplace (Table 6). The univariate binary logistic regression model showed that caesarean delivery (OR 7.4; 95% CI 2.22, 24.65), duration of maternity leave more than 120 days (OR 5.19; 95% CI 1.00, 26.94), and occupation as a doctor, dentist, or nurse (OR 4.08; 95% CI 1.33, 12.50) were the likely successful predictors affecting exclusive breastfeeding (Table 7). Multivariate analysis was used to control and assess the independent effects. Our study found that caesarean delivery (OR 7.9; 95% CI 2.00, 31.08) was the only independent variables affecting exclusive breastfeeding (Table 8).

**Table 6:** Factors in bivariate analysis associated with exclusive breastfeeding (*N* = 65)

Variable	EBF (*n* = 43)	No EBF (*n* = 22)	*p* value
Demographic data of mothers and their infants			
Parity			0.082
1	26 (59)	18 (41)	
More than 1	17 (81)	3 (19)	
Educational level			0.842
High school	5 (71)	2 (29)	
Bachelor’s degree	31 (67)	15 (33)	
Master’s degree or higher	7 (58)	5 (42)	
Income (Baht per month)			0.269
Less than 20,000	8 (57)	6 (43)	
20,001–50,000	21 (62)	13 (38)	
More than 50,000	14 (82)	3 (18)	
Delivery type			0.001**
Vaginal delivery	6 (33)	12 (67)	
Cesarean delivery	37 (79)	10 (21)	
Type of birth hospital			0.349
Public	32 (63)	19 (37)	
Private	11 (79)	3 (21)	
Work information			
Maternity leave			0.022**
Less than 90 days	18 (62)	11 (38)	
91–120 days	8 (47)	9 (53)	
More than 120 days	17 (90)	2 (10)	
Occupation			0.011**
Doctor/dentist/ nurse	26 (81)	6 (19)	
Other	17 (52)	16(48)	
Number of working hours per week, median (interquartile range)	40(40–46)	45(40–50)	0.408
Average stress scores (range 1–5), mean (SD)	3.14 (1.21)	3.55 (1.14)	0.196
Participant responses regarding workplace and breastfeeding			
There are policies that support breastfeeding in the workplace	13 (72)	5 (28)	0.522
There are breastfeeding specialists or consultation teams in the workplace	14 (88)	2 (12)	0.038**
Appropriate duration of maternity leave	33 (66)	17 (34)	0.962
The workplace has a breastfeeding room	5 (55)	4 (45)	0.473
The workplace has a refrigerator used for storing pumped milk	15 (63)	9 (37)	0.634

*EBF* Exclusive breastfeeding, *SD* Standard deviation, ** *p* value < 0.05

**Table 7:** Univariate binary logistic regression analysis to determine the factors associated with exclusive breastfeeding (*N* = 65)

Variable	EBF (*n* = 43)	No EBF (*n* = 22)	*p* value	OR (95% CI)
Delivery type				
Vaginal delivery	6 (33)	12 (67)	Ref	
Cesarean delivery	37 (79)	10 (21)	0.001**	7.4 (2.22, 24.65)
Maternity leave				
Less than 90 days	18 (62)	11 (38)	Ref	
91–120 days	8 (47)	9 (53)	0.324	0.54 (0.16, 1.83)
More than 120 days	17 (90)	2 (10)	0.050**	5.19 (1.00, 26.94)
Occupation				
Doctor/dentist/ nurse	26 (81)	6 (19)	0.014**	4.08 (1.33, 12.50)
Other	17 (52)	16(48)	Ref	
There are breastfeeding specialists or consultation teams in the workplace	14 (88)	2 (12)	0.052	4.83 (0.99, 23.61)

*EBF* Exclusive breastfeeding, *OR* Odds ratio, *CI* Confidence interval, ** *p* value < 0.05

**Table 8:** Factors in multivariate analysis associated with exclusive breastfeeding (*N* = 65)

Variable	EBF (*n* = 43)	No EBF (*n* = 22)	Crude OR (95% CI)	Adjusted odds ratio (95% CI)
Delivery type				
Vaginal delivery	6 (33)	12 (67)	1	1
Cesarean delivery	37 (79)	10 (21)	7.4 (2.22, 24.65)	7.9 (2.00, 31.08) **
Maternity leave				
Less than 90 days	18 (62)	11 (38)	1	1
91–120 days	8 (47)	9 (53)	0.54 (0.16, 1.83)	0.4 (0.09, 1.54)
More than 120 days	17 (90)	2 (10)	5.19 (1.00, 26.94)	3.5 (0.51, 23.77)
Occupation				
Doctor/dentist/ nurse	26 (81)	6 (19)	4.08 (1.33, 12.50)	2.7 (0.69, 10.54)
Other	17 (52)	16(48)	1	1

*EBF* Exclusive breastfeeding, *OR* Odds ratio, *CI* Confidence interval, ** *p* value < 0.05

Regarding those factors associated with discontinuing breastfeeding after returning to work (Table 9), 50 participants were still breastfeeding when they returned to work. Nearly half (42%) of women had to stop breastfeeding within six months after returning to work. There were no significant differences in the associated factors between the two groups. Sixty-seven percent of doctors, dentists, and nurses were able to continue breastfeeding whereas only 48% of those with other occupations were able to do so (*p* = 0.179). There was some evidence that more participants with previous lactation experience (75%) were able to continue breastfeeding compared with those who had no lactation experience (50%) (*p* = 0.095). The average stress score was 2.97 in the group that continued breastfeeding successfully and 3.43 in the group that did not (*p* = 0.194).

**Table 9:** Factors in bivariate analysis associated with continued breastfeeding after returning to work (*N* = 50)

Variable	Continued breastfeeding (*n* = 29)	Stopped breastfeeding within 6 months (*n* = 21)	*p* value
Maternity leave			0.169
Less than 90 days	11 (48)	12 (52)	
91–120 days	9 (82)	2 (18)	
More than 120 days	9 (56)	7 (44)	
Occupation			0.179
Doctor/dentist/nurse	18 (67)	9 (33)	
Others	11 (48)	12 (52)	
Number of working hours per week, median (interquartile range)	40(40–45)	43(40–45)	0.992
Previous lactation experience	12 (75)	4 (25)	0.095
Anatomical breast problems	8 (67)	4 (33)	0.485
Parental assistance during maternity leave	20 (57)	15 (43)	0.851
Supported by family members	27 (56)	21 (44)	0.503
Method of breastfeeding during maternity leave (*n* = 63)			0.255
Direct breastfeeding	26 (62)	16 (38)	
Breast pumping only	3 (38)	5 (62)	
Average stress scores (range 1–5), mean (SD)	2.97 (1.15)	3.43 (1.33)	0.194

*SD* Standard deviation

### Results of focus group discussion (FGD)

In the FGD, seven participants were still breastfeeding before returning to work. Four were able to continue breastfeeding whereas three are unable to do so. The profile of study participants is shown in Table 10. Four major themes and subthemes were identified from the data analysis, which were personal–social factors and workplace-associated factors.

**Table 10:** Profiles of study participants in focus group discussion

Successful continuation of breastfeeding after returning to work	Participant	Duration of maternity leave (months)	Position	Number of children	EBF for 6 months
Yes	1	2	Doctor	2	Y
	2	9	OPD nurse	1	Y
	3	3	General administration officer	2	Y
	4	3	ICU nurse	1	Y
No Stopped BF within 6 months	5	3	General administration officer	1	N
	6	9	Doctor	1	N
	7	3	Pharmacist	2	Y

*EBF* Exclusive breastfeeding

### Personal–social factors: supporting factors

#### Direct breastfeeding

Some participants discovered that direct breastfeeding promotes a longer duration of breastfeeding by reducing exhaustion from breast pumping and direct stimulation from the baby’s sucking, leading to more effective milk production. Additionally, when returning to work and being unable to continue pumping milk, the baby receives breast milk directly from the mother as long as they nurse.

*“With breast pumping at work, the amount [of milk] decreased because pumping does not match natural stimulation from the child’s sucking like when at home, so the amount of breast milk was less.” (ID 6)*

*“After that, I feel much better because with direct breastfeeding, I don’t have to pump for breast milk, this saves a lot of energy. Without having to use the machine, I feel a lot happier with breastfeeding. On top of that, I feel lucky cause my child was able to direct breastfeeding very well, I mean my child can feed directly from my breast and this causes the milk to produce well and there is enough breast milk to feed him continuously” (ID 2)*

#### Support from healthcare personnel

##### Lactation clinic

The role of breastfeeding clinics can lead to more successful breastfeeding. Choosing to give birth in a hospital that supports breastfeeding is the key factor for a good start to breastfeeding. In addition, proper advice on breastfeeding after the mother has returned home and positive attitudes from the staff in the clinic will encourage the breastfeeding mother.

*“I gave birth at [ . . . hospital name]. There was no lactation clinic there, Hospitals are actually very important, and [ . . . hospital name] doesn’t have a lactation clinic. They let me leave when I did not even know how to nurse, I don’t know how to breastfeed. When I got home, I had breast engorgement problems and mastitis.” (ID1)*

*“The lactation clinic at [ . . . hospital name] is really good, I must say, the place is really good, it’s like heaven-sent. Only 50 Baht per day and I get to stay there for the whole day. They really support us; they wait for us. I bring my bags over to stay with them from 8 AM to 4 PM . . . for 2 weeks. They stay with me the whole time, never abandon me, and stay with me to make sure that I know how to do it.” (ID 1)*

*“I went to a lactation clinic at [ . . . hospital name] around three times. Luckily, I have a friend who works at the unit, so I received several lessons and learned different techniques. I felt more confident to breastfeed.” (ID 4)*

#### Support from family members

##### Psychological support from family members

Family support affects the breastfeeding success rate. Some participants with unsupportive family members do not know how to cope with this issue.

*“Not to brag or anything, but my husband is knowledgeable about breastfeeding; he told me to breastfeed as long as possible.” (ID 4)*

*“My obstacles are that, first, my family members don’t understand. I don’t know why they don’t understand that breastfeeding is important. It is difficult. There was no support; my husband did not support me. He was like, ‘if the child doesn’t take your breast, then use formula instead.’ Yeah, he is my husband, but I’m strong enough to ignore him.” (ID 1)*

##### Babysitting by family members

Having someone take care of the baby during maternity leave can increase the breastfeeding success rate because this can reduce maternal tiredness. Mothers have enough time to pump milk while having someone take care of the baby.

*“My husband is working from home most of the time, so he has time to help take care of the child even better than I do. I kind of have the know-how so when I need to breastfeed, I breastfeed; when I need to breast pump, I do it. He can take care of the child when I have to breast pump.” (ID 4)*

*“The main obstacles are that my husband needs to go to work and I have to take care of the child by myself during the day until the evening when my husband returns. Yes, it’s a little exhausting. When I finish breast pumping, cleaning up, and getting everything done, then my child is already up. I have to continue this routine; it’s like I’m alone in this. Fortunately, my grandmother has helped me out for around 4 months. With this, I have time to do something else because my grandmother helps out.” (ID 2)*

### Personal–social factors: structural barriers

#### Breast problems

Some women reported that breast problems are an important obstacle to successful long-term breastfeeding because this can result in inefficient breastfeeding.

*“I can’t direct nurse at all because of a nipple problem.” (ID 5)*

#### Self-confidence in knowledge and skill in breastfeeding

Some participants felt unsure about their knowledge and skills.

*“When I got home, I had to admit that I had no experience in breastfeeding, but my child was good at sucking, with energy too. Before giving birth, I was assessed and told everything was ok, but when I got home, I have to say—truly with my own judgement—I feel like when I look at myself without experience, I am not so confident. My child did not gain weight and looked yellow. I also kept track of the amount of pees and poos; I was not sure whether it was enough. In the end, I decided to use the formula early on.” (ID 6)*

#### Impact of COVID-19

The situation during the COVID-19 pandemic has caused breastfeeding to be more difficult in several ways.

##### Hospital unable to provide full service, especially lactation clinics

One participant had to change the hospital where she had intended to give birth

*“I gave birth during the first wave of the COVID-19 outbreak. At first I received my prenatal care at [ . . . hospital name], but at the time, the operating room was closed. So I had to give birth at a private hospital where there was no lactation clinic. I wanted to go to the clinic but with the outbreak. . . and my husband’s family did not want me to go to the clinic because hospitals were risky areas.” (ID 6)*

##### Domestic transportation services affected

This makes it impossible to send expressed breast milk to a child who lives in a different area.

*“Public buses did not run [owing to the COVID-19 outbreak]. I cried because I did not know what to feed my child with.” (ID 3)*

##### Pumping milk outside the home is difficult

This was because of concerns about cleanliness during the pandemic, especially in a hospital-type workplace

*“With the COVID-19 outbreak, I could not breast pump anywhere. I feared getting infected; hospitals are risky areas. I feel it is unsafe to breast pump anywhere—which clinics to go to? Is it hygienic enough? I was concerned.” (ID 2)*

##### Workloads

During the COVID-19 pandemic, health care workers had greater workloads, and they needed to work harder,

*“When I had to work, I was stressed out, so the amount of breast milk gradually decreased.” (ID 2)*

##### Vaccines and drugs

Breastfeeding mothers were not confident about receiving the COVID-19 vaccine or new COVID-19 drugs because of breastfeeding safety.

*“I did not believe in the vaccine against COVID-19. I have to receive the vaccine, but I was unsure about breastfeeding, so I stopped. When I was injected with the vaccine, I stopped breastfeeding. The relatively new vaccine was an inactivated type, and my husband had a feeling he did not want to risk it with the child.” (ID 6)*

### Other personal–social factors

#### Maternal attitudes toward breastfeeding and prenatal breastfeeding intention

All participants had a positive attitude toward breastfeeding, but it was not enough to achieve breastfeeding in the long term because of other important factors, such as a lack of support.

*“I was strongly determined before gave birth that I want to breastfeed as long as I could, I find a lot of information, but I ended up unsuccessfully.” (ID 6)*

*“I also realized the importance of breast milk, so I tried to do everything I could, like eating some leeks, because someone said that it would help, I did everything that grandmother recommended and last it was not work and I gave up.” (ID 5)*

#### Previous maternal breastfeeding experience

This may not be sufficient to guarantee breastfeeding success because there are various kinds of breastfeeding problems in different lactations.

*“With the second pregnancy, I thought I was doing well. I was confident, but I still failed because there are millions of problems with mothers’ breast milk. It was breast engorgement with the first pregnancy, then the sore nipples with the second.” (ID 1)*

### Workplace-associated factors: supporting factors

#### Adequate maternity leave

Sufficient maternity leave results in a longer duration of breastfeeding, especially in those who have difficulty pumping milk at work, because some mothers can store enough breast milk expressed during maternity leave. However, in some circumstances, maternity leave was not practical owing to the culture of the workplace or the nature of some professional occupations.

*“A supporting factor was that I was able to take a prolonged leave.” (ID 6)*

*“I must say that [with my profession], generally, I can’t take leave longer than 3 months. Leave without pay is difficult because this requires the supervisor’s understanding. I was lucky because everyone at my unit has a child and I’m the youngest. My supervisor was understanding, and my pregnancy was during the COVID-19 outbreak. My supervisor saw my determination to breastfeed and allowed me to take prolonged leave. This was helpful; otherwise, my child would only have gotten 3 months of breast milk. With the outbreak, I could not breast-pump anywhere.” (ID 2)*

#### Childcare at the workplace

During breaks, mothers can bring expressed breast milk directly to their child or nurse them. However, the main problem is limited availability. Most employees were unable to send their children to childcare.

*“Let’s just say that the childcare here is excellent. When my child was younger, I came here to breastfeed during lunchtime.” (ID 1)*

*“The problem was about babysitters; I raised the second child myself. The childcare was full, so I had to hire outside help; then, the fee was higher than that of childcare. The standard was different too.” (ID 7)*

#### Positive attitudes of colleagues and managers

Positive attitudes and personal experiences of breastfeeding among colleagues and managers would promote the success of breastfeeding after returning to work. Colleagues and employers with previous breastfeeding experience usually understand the context of the mother who must take a break to pump milk every 3–4 h. However, attitude alone is not sufficient without a clear organizational policy, such as a breastfeeding break for employees.

*“I was lucky in terms of the situation. Colleagues at my unit were pregnant before and they breast pumped quite extensively. When I came back to work, I was trying to breast pump too. On a less busy day, I tried to breast pump on time by having my colleagues help me and look after patients briefly while I was expressing milk.” (ID 6)*

*“I was lucky to be pregnant while working with this unit and gave birth around the same time. With the first child, we took turns to breast pump. Most colleagues have kids, so they understand about breastfeeding.” (ID 4)*

*“Some colleagues understand while some don’t. When we take the time, like for 30 min but every 3 h, it may affect some people.” (ID 1)*

*“In terms of supervisors, they were supportive. But with the continuous nature of the work without assigned break time, I did not want to take the opportunity. For example, you can go for 30 minutes or an hour. It kind of puts the pressure on. Colleagues understand, but the nature of the work does not really allow [for it].” (ID 7)*

### Workplace-associated factors: structural barriers

#### No private facilities (location and equipment) for expressing and storing breast milk

The absence of a breastfeeding room in the workplace is an important barrier for working mothers who need to continue pumping breast milk after returning to work. All participants strongly agreed about this issue, especially with their type of work in the hospital, which is concerned with cleanliness. Some participants mentioned that in a multi-campus area, the organization should provide a breast pumping facility on all campuses. The facilities include separate rooms and a refrigerator for storing milk.

*“That area is definitely not hygienic because patients interact there all the time, and it’s like [ID1] and everyone said, there are no rooms (facilities) for us.” (ID 2)*

*“The room at the back has one unoccupied refrigerator, but there are other items inside too, so there may be contamination. But I secretly put my stuff in the freezer; when the work or meeting is over at 4 PM, I take it out.” (ID 3)*

#### Lack of breastfeeding break

Most participants could not breast pump during work hours because of the continuous nature of the work, especially caretaker work with no assigned breaks. Office-based work with clear breaks was better for allocating time to breastfeeding compared with caretaker positions.

*“I feel like when I have the time to breast pump. I have so many patients to take care of. If I leave for 30 minutes, that means 30 minutes of work. I could draw blood samples from many patients. This will waste time, and so many things need to be done. I felt like I couldn’t continue to breastfeed, and couldn’t breast pump either, so I decided to use bottled milk. Plus, I have to draw blood samples or work at a counter and move around all the time.” (ID 2)*

*“Maybe I’m lucky cause I work in the back office, maybe luckier than others who work directly with patients. Lunchtimes are office hours, so we have time to rest.” (ID 3)*

*“The intensive care unit is always busy, and I have to perform treatments in patients. Inevitably, you can’t take a break to express breast milk like everyone else. After getting back to work, I cannot pump as scheduled or take a long period for pumping because our patients are in critical condition and need care. When working, work is work, and [expressing milk] becomes an obstacle.” (ID 4)*

*“If I take a prolonged break, I’m not sure if that’s considered taking advantage of others, so my initial intention of breastfeeding for a year has decreased to just 8 months because I kept some stocks. But the stocks were not enough because I couldn’t breast pump enough later when I worked. So, I didn’t have enough breast milk for my child. It was not as planned, so I lost the determination to continue; it was a struggle.” (ID 7)*

#### Inconvenient uniform for pumping

Thai nurse uniforms are obstacles to breast pumping. Comfortable outfits, such as medical scrubs, make breast pumping easier and compatible with supporting devices such as a hands-free pump.

*“I couldn’t breast pump at the hospital because our uniforms and the nature of our work do not allow the time for a scheduled breast pumping session. Uniforms can’t just be draped over the shoulder; they really can’t. We will be looked at like, ‘What’s wrong with you? Why do you have to wear that?’ If we wear that to work or to draw blood samples from patients, they will look at us strangely too.” (ID 2)*

*“Features of the ICU uniforms may be a little better than what [ID2] said because I didn’t wear a nurse’s uniform. No blouse or skirt; we wear an outfit. Actually, it should be like this for every unit, right? Just a top and pant.” (ID 4)*

### Other workplace-associated factors

#### Persistent attempts

Mothers who try to solve problems by themselves are an important factor for successful and continuous breastfeeding at work, even when the work environment or policy is not supportive. Solutions include a change in work schedule to facilitate breastfeeding and use of supporting devices such as a hands-free pump. Every mother who succeeded at continued breastfeeding talked about the great efforts needed to overcome obstacles.

*“Obstacles are no longer obstacles because I can do this anywhere and anytime and on time. My three ounces of breast milk can feed my child all day and night. The day when I stopped breastfeeding was only when I was tired. In the end, I have to find the way to keep up; it is on the mother to struggle and find the way, find the pump, find the way to keep up.” (ID 1)*

#### Requirements

FGD participants discussed the private facilities needed for expressing and storing breast milk, which include the following.

- Breastfeeding room with necessary breast pumping equipment, such as a refrigerator for storing expressed breast milk, breast pumping equipment, milk storage bag, and ice pack to transfer milk.
- Some participants mentioned that this room should provide storage service for expressed milk so that those who do not have their child close by can transport the milk directly from here.
- A lactation consultant for employees and a breastfeeding clinic.
- Community peer support or a network of women to support continued breastfeeding at work.
  *“Having a support group helps us go on when we get to talk, chat, and share, whether positive or negative. Another important thing is that we need a nurse or someone who has knowledge or experience to guide us because if done in the wrong way, lactation can be painful and bring tears, as everyone said. Those who have experience could help and share. Sharing experience is helpful.” (ID 1)*
  *“When we share opinions and experience, this helps new moms or less experienced moms learn something new. Sharing while pumping is relaxing too.” (ID 3)*

## Discussion

In this study, we attempted to identify supporting factors and barriers in continuous breastfeeding at a hospital-type workplace. Although the definition of exclusive breastfeeding in this study is stricter than that of WHO, which is captured in only a 24-h period prior, the rate of EBF among the included hospital employees was 66%, which is higher than the average EBF rate in Thailand [5]. This result is consistent with the previous study that female healthcare workers have higher breastfeeding rates than those general population [22]. Currently, the worldwide caesarean section rates had been increasing [23]. Our study found that the caesarean section rate was 72% which was higher than average caesarean section rate in Thailand [24]. One of the reasons may be that our study collected the data from participants who gave birth at both public and private hospitals, in contrast to previous studies which mostly collected data in the public hospitals alone. A previous study in Thailand found that the rate of caesarean section in the private hospitals tend to be higher than in the public hospitals [25]. Compared with vaginal delivery, caesarian delivery may negatively impact breastfeeding initiation, milk production, and duration of exclusive breastfeeding [26]. However, our study found that the rate of EBF in the caesarian delivery group was higher than that in the vaginal delivery group (78% vs. 33%, (OR 7.9; 95% CI 2.00, 31.08, *p* = 0.001). Our findings are contrary to most studies and are possibly due to the small sample.

Feeding directly from the breast promotes a longer period of breastfeeding. With effective latching on, mothers are not only more confident that their children are adequately nourished but they are also less fatigued from pumping breast milk around the clock [27]. Additionally, feeding directly at the breast is more natural and less painful than pumping [28], and the baby continues to receive breast milk directly from the maternal breast as long as they nurse. In particular, mothers who can bring their children to work and directly breastfeed during breaks can maintain breastfeeding longer [29]. This study further supports that having childcare in the workplace is likely to improve breastfeeding success among employees.

The previous meta-analysis showed the rate of breastfeeding after returning to work is about 25% which is widely heterogeneous across the world, ranging from 2% to sixty one percent. Interestingly, both economic status and cultural aspect play an important role in the continuation of breastfeeding [30]. The result of our study found that about 76% (50/65) of mothers were continuing to breastfeed before returning to work but 24% (12/50) had to stop breastfeeding within three months after returning to work. The breastfeeding rate after returning to work in our study seems to be slightly higher than the result from that meta-analysis, this may be caused by a heterogenicity of the duration of returning to work after birth, which ranged from 1 to 12 months in the meta-analysis [30].

The duration of maternity leave is a key factor in sustained breastfeeding [31] especially paid maternity leave [32]. Although our study did not find any statistically significant difference in the longer maternity leave duration(*p* = 0.05), this could be a result of the small sample size. In low- and middle-income countries, each legislated month of additional paid maternity leave would lead to a 2.2-month increase in breastfeeding duration [33] and reduce infant deaths by nearly 8 per 1,000 live births [34]. Currently, Thai law offers paid maternity leave for just 98 days [7]. Therefore, other solutions may improve the success of breastfeeding, such as allowing women to return to work in a variety of ways, including teleworking or working part-time [35]. It has been discovered that part-time employment increases the length of breastfeeding and the acceptance of remote working in the COVID-19 pandemic may also support breastfeeding [30].

According to the FGD, in some circumstances, maternity leave was not practical because of the workplace culture or the nature of some professional occupations. So, the design of the workplace plays an important role in promoting breastfeeding [32]. The factors involved include a breastfeeding break, facilities and equipment for breast pumping and breastfeeding, as well as support from colleagues and supervisors [36]. All participants in the FGD strongly agreed on this topic, especially with their type of work in a hospital where cleanliness is of great importance. Workplace breastfeeding rooms are inexpensive and should be provided to improve breastfeeding [37]. Lack of proper breastfeeding facilities at work was almost double the discontinuation rate of breastfeeding [38]. Further, most participants in the FGD could not breast pump during working hours because of the continuous nature of the work, especially healthcare workers. Job characteristics is another factor in continuous breastfeeding. Breastfeeding break times are designed for nursing mothers working in office-based settings more so than for mothers working in service-based settings because of more flexible work hours in the former group [27]. Therefore, clear and appropriate breastfeeding breaks for all types of job position are needed. One study found that only two breaks during the workday for breast pumping is enough and can increase the chances of successful breastfeeding at work [39]. Further, employees who had adequate break time increased threefold of EBF at six months compared to those without sufficient breastfeeding break [40].

Participants in the FGD also focused on the attitudes of colleagues and supervisors. Positive beliefs and values regarding breastfeeding is a main supporting factor in continued breastfeeding among mothers after returning to work. Moreover, supervisors with previous experience of breastfeeding usually understand the context of a mother who must take a break to pump milk every 3–4 h. According to a systematic review in 2021, the data from most studies has shown that support from coworkers or supervisors did not have a significant association with the duration of breastfeeding, but it could be an indirect impact on prenatal breastfeeding confidence. In addition, when combined with other strategies to promote breastfeeding at the workplace, it showed an increase odd of continue breastfeeding after returning to work [18].

However, attitudes alone are not enough for successful continued breastfeeding without a clear organizational policy. All the above factors, including a breastfeeding room, breastfeeding break, and positive attitude toward breastfeeding, derive from the organization’s breastfeeding policy [18, 41]. Evidence suggests that interventions given in a variety of settings, including communities, health systems and workplace can increase breastfeeding rate among mothers who must return to work during the recommended period of breastfeeding. This would create a supportive breastfeeding environment, which is an important factor in continuous breastfeeding [42]. Nevertheless, organizational and interpersonal change are also required to sustain breastfeeding in working conditions [18]. Moreover, enhancing breastfeeding rate can be accomplished through expanding successful interventions, legislation, and programs in terms of country-specific best practices. However, inadequate maternity protection is the significant problem in several countries which should be addressed immediately [43].

Interestingly, one barrier in nursing was found to be the uniform worn by healthcare workers. A female police officer in the United States was forced to quit her job because wearing a tight-fitting bulletproof vest put her in danger of breast infections and reduced milk production [44]. Thus, employers should consider the special needs of breastfeeding employees, such as an appropriate uniform or workwear for particular groups.

Under conditions of the COVID-19 pandemic, breastfeeding has become more difficult in many ways, especially among healthcare workers who have had heavy workloads. A study in the United Kingdom (UK) found both positive and negative impacts of COVID-19 on breastfeeding. Nearly half of mothers in the UK who completed an online survey reported feeling better about breastfeeding during lockdowns because they had more time at home, were less stressed, and had fewer visitors. In contrast, approximately one-fourth of respondents reported challenging situations owing to a lack of support and mental stress, which led to stopping breastfeeding early [45]. Additionally, although the WHO recommends that breastfeeding mothers can continue breastfeeding after receiving a COVID vaccine [46], many nursing mothers do not feel confident about the safety of vaccines and new drugs while breastfeeding, which is also a primary barrier to continued breastfeeding after receiving a COVID vaccine.

This study was strengthened by the use of a mix method study, which can simultaneously gather quantitative and qualitative data, obtained the benefits and the viewpoints of both approaches. From the quantitative results, most of the participants had a good attitude toward breastfeeding. We found that 90% of participants intended to EBF for six months, and more than 3/4 of participants were able to continue breastfeeding before they returned to work. However, nearly 1/4 of them discontinued breastfeeding within three months after returning to work. Their main reasons reported in the questionnaires were the lack of a policy to support breastfeeding, experts in counselling, and facilities for pumping and milk storage. Therefore, we followed up on these workplace-associated factors in terms of the workplace’s existing supporting factors and structural barriers qualitatively via focus group discussion.

For workplace factors, although employees could have only 3-month-maternal leave, most of the participants agreed that was not sufficient. However, the attitudes of colleagues and supervisors mitigate this limitation. Some participants discussed that their supervisors allowed extended leave without pay which normally depended on the supervisor’s judgement. In addition, considerate colleagues play an important role in subsidizing extra workload. This emphasizes that advocacy for longer maternal leave at the national level and promoting considerate workplace culture are crucial. Some hidden issues were discovered by focus group discussions, such as a nursing uniform and lack of regular breastfeeding breaks were found to be an obstacle to breast expression. Although our participants in the FGD came from the successful and unsuccessful continuous breastfeeding, all of them initially had a strong willingness to continue breastfeeding after returning to work which is a characteristic of our interest. On the other hand, the heterogenicity of the participants may allow for contrasting viewpoints. The variation of participant experiences would help in designing further breastfeeding support in the workplace. Our study also had some limitations. First, it was a relatively small sample size even though we had recruited all woman employees who having maternity leave during that period. This small size of sample might not have enough power to find some significant factors which affect in the continuation of breastfeeding. Second, due to some specific reasons, only having one focus group discussion can potentially be the limitation of this study also. Last, this study was conducted during the COVID-19 pandemic, so the FGD had to conduct via online meeting. Online focus group might have some barriers such as the difficulty for the researchers to observe the non-verbal communication of the participants.

## Conclusions

The findings of study are crucial in determining how to best support employees who are struggling with breastfeeding after returning to work, particularly during the ongoing COVID-19 pandemic. Although hospital employees are more likely to succeed in breastfeeding, particularly healthcare workers, enormous time commitment and effort are required to sustain breastfeeding after a return to work. Our study confirms the critical role of the organization in providing support in all areas pertaining to support for nursing mothers. We recommend that breastfeeding-friendly policies should be implemented in the workplace, with clear operating guidelines such as providing a breastfeeding room, breastfeeding break, childcare at the workplace, and appropriate uniforms for nursing mothers, as well as the organization advocating positive attitudes toward breastfeeding, to sustain breastfeeding. Additionally, much can be learned from others who have had positive experiences to ensure that all breastfeeding women are better supported in the workplace in future.

## Data Availability

The datasets used and analyzed in the current study are available from the corresponding author on reasonable request.

## Abbreviations

EBF: Exclusive breastfeeding
FGD: Focus group discussion
GA: Gestational age
UN: United Nations
UNICEF: United Nations Children’s Fund
WHO: World Health Organization
